# Genetic characterization of HIV-1 epidemic in Anhui Province, China

**DOI:** 10.1186/s12985-020-1281-y

**Published:** 2020-02-03

**Authors:** Dong Zhang, Jianjun Wu, Yu Zhang, Yuelan Shen, Sheying Dai, Xiaolin Wang, Hui Xing, Jin Lin, Jingwan Han, Jingyun Li, Yizu Qin, Yongjian Liu, Lifeng Miao, Bin Su, Hanping Li, Lin Li

**Affiliations:** 1 Department of AIDS Research, State Key Laboratory of Pathogen and Biosecurity, Beijing Institute of Microbiology and Epidemiology, 20 Dongda Street, Fengtai District, Beijing, 100071 China; 2 Anhui Provincial Center for Disease Control and Prevention, Hefei, 230601 China; 3 State Key Laboratory for Infectious Disease Prevention and Control, National Center for AIDS/STD Control and Prevention, Chinese Center for Disease Control and Prevention, Beijing, 102206 China

**Keywords:** HIV, Subtypes, Transmission network, Transmitted drug resistance

## Abstract

**Background:**

Anhui Province in China is facing a severe HIV epidemic with an increasing number of newly diagnosed cases.

**Methods:**

In this study, HIV genetic characteristics in the province were investigated. Newly reported HIV-positive individuals from 15 districts of Anhui Province were enrolled and interviewed. Total viral RNA was extracted from plasma isolated from blood samples. We amplified and sequenced an HIV *pol* fragment of the 1062 bp. The sequences were used for determination of HIV subtypes and the presence of drug resistance mutations. Transmission networks were constructed to explore possible relationships. And all of assembled partial pol genes were submitted to the Stanford HIV Drug Resistance Database website to find the transmitted drug resistance.

**Results:**

Partial *pol* gene sequences were obtained from 486 cases. The results showed that MSM was the most dominant transmission route (253, 52.06%), followed by heterosexual transmission (210, 43.21%) and blood-borne transmission (1, 0.21%). Many subtypes were identified, including CRF01_AE (226, 46.50%), CRF07_BC (151, 31.07%), subtype B (28, 5.76%), CRF08_BC (20, 4.12%), CRF55_01B (15, 3.09%), CRF68_01B (7, 1.44%), CRF67_01B (3, 0.62%), CRF57_BC (2, 0.41%), CRF59_01B (2, 0.41%), CRF79_0107 (2, 0.41%), subtype C (2, 0.41%), CRF64_BC (1, 0.21%), and circulating recombinant forms (URFs) (27, 5.55%). Four transmission subnetworks containing high transmission risk individuals (with degree ≥4) were identified based on CRF01_AE and CRF07_BC sequences, including two CRF01_AE transmission subnetworks constituted by elderly people with average ages of 67.9 and 61.5 years. Infection occurred most likely through heterosexual transmission, while the other two CRF07_BC transmission subnetworks consist mainly of MSMs with average ages of 31.73 and 34.15. The level of HIV-transmitted drug resistance is 3.09%.

**Conclusions:**

The simultaneous spread of multiple HIV subtypes in Anhui province underscores that close surveillance of the local HIV epidemic is necessary. Furthermore, the elderly people were frequently involved, arguing for behaviour intervention in this specific population besides the MSM risk group.

## Introduction

The first HIV case was reported in China in 1985 [1]. Since then, HIV-1 has spread widely in the country. As of August 31, 2018, 841,478 people living with HIV were reported nationwide. Among them, 348,223 cases were AIDS patients [2]. The prevalence of HIV-1 has become a tremendous challenge to public health in China.

**Fig. 1:**
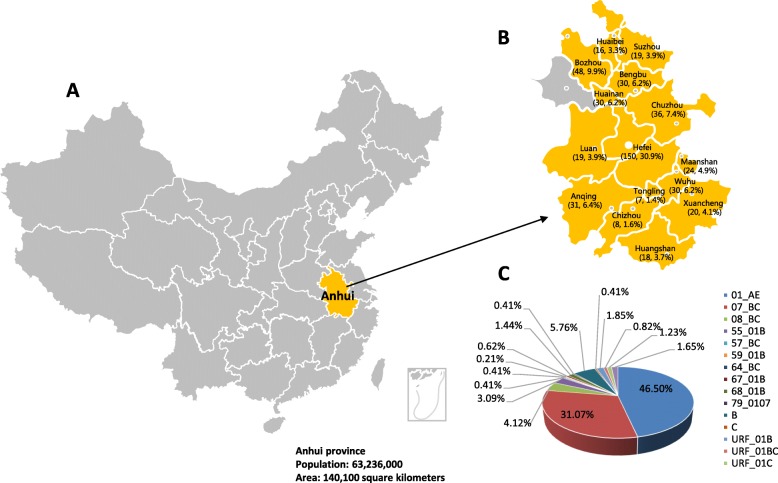
Geographical distribution of HIV-1 subtypes in Anhui Province. The study area is highlighted on the map of China (Fig. 1**a**). The distribution of HIV-1 samples in each district across Anhui Province (*n* = 15), except Fuyang, is illustrated in the inset (Fig. 1**b**). The proportions of each subtype are presented in the pie chart (Fig. 1**c**). The land area and the population information of Anhui Province are listed at the bottom

Anhui Province plays an important role in the HIV-1 epidemic, where many HIV infections have been identified in former commercial blood donors [3] (Fig. 1a). The first HIV-positive case was discovered in Anhui Province at the end of 1994. The number of HIV cases subsequently increased quickly in the whole province. As of October 31, 2018, the total number of HIV-positive individuals in the province reached 17,183. In addition, the main transmission route of HIV has also changed. The proportion of newly diagnosed individuals infected by HIV through homosexual contact has rapidly increased in recent years [4] and has become the dominant transmission route, accounting for 98.32% of sexual transmission. The MSM population in China is characterized by high mobility, which contributed greatly to the spread of HIV in different areas [5]. Furthermore, MSM always play a bridge role in HIV transmission among different populations in China [6]. The key role that the MSM population plays in HIV prevalence rationalizes the study of HIV characterizations in the MSM population in Anhui Province.

In a previous study, we performed a comprehensive investigation of the HIV epidemic in the Fuyang District of Anhui Province [7]. In this study, we characterized the HIV epidemic in other districts of Anhui Province.

## Material and methods

### Study population and samples

In this study, a total of 518 individuals who were confirmed as HIV antibody positive from October 2017 through August 2018 in 15 districts of Anhui Province were enrolled. Demographic information was collected with informed consent [8]. The distribution of the cases in different districts is presented in Fig. 1b.

### Sequence data

Total viral RNA was extracted from plasma isolated from blood samples using the Roche High Pure Viral RNA Kit (Roche, REF: 11858882001). A nested reverse transcriptase polymerase chain reaction (RT-PCR) was used to amplify a *pol* fragment of the 1062 bp length region (nucleotide 2253–3314 by using HXB2 as calibrator) spanning the protease gene and partial reverse transcriptase gene using the One Step RNA PCR Kit (Takara, RR055A) and the ExTaq Kit (Takara, RR902A) with primer sets and thermal cycling conditions as described previously [9]. The PCR products were purified and subjected to direct DNA sequencing on an Applied Biosystems 3730 Sequencer.

### Identification of subtypes

HIV partial *pol* genes were successfully obtained from 486 samples. All assembled sequences were submitted to the HIV-1 Sequence Quality Control Tool (https://www.hiv.lanl.gov/content/sequence/QC/index.html) to confirm the sequence quality. All of the gene segments contained correct open reading frames (ORFs). Blast and PIP analyses were also used in the Quality Control Tool to identify the subtype of each sequence. Simultaneously, the sequences were submitted to REGA, the tool designed to use phylogenetic methods to identify the subtype of a specific sequence [10]. A phylogenetic tree was constructed by the neighbour-joining method based on the Kimura 2-parameter model with 1000 bootstrap replicates in MEGA6 software [11]. The reference gene sequences of subtypes B, CRF01_AE, CRF07_BC, CRF08_BC, CRF67_01B, and group O were downloaded from the HIV database (https://www.hiv.lanl.gov/content/index). The results from the software were finally combined to determine the subtype of each sequence.

### Construction of the transmission network

The HIV transmission network was constructed based on the similarity of HIV sequences [12], which can be used to explore relationships of different strains. The aligned sequences were entered into Hyphy 2.2.4, and the genetic distances between sequences were calculated based on the TN93 [13] (Tamura-Nei 93) model. The genetic distance among sequences belonging to the same subtype was set up as the cutoff value for transmission network construction, which maximized the number of clusters in the network and avoided the formation of a giant cluster. The networks were then processed and visualized using Cytoscape v3.3.0 software [14]. The HIV transmission network consists of two basic elements: nodes and edges. Nodes represent an HIV sequence or an HIV-infected person, and the edge represents two HIV-infected people connected with a potential transmission relationship, that is, the gene distance between the two sequences is lower than the set threshold. The subnetworks represent the groups composed of at least two nodes in the network. The degree of each node means the number of edges connecting to it, which represents the potential transmission partners of the node. Oster et al [15] divided the study population into three categories based on the number of degrees: 0, 1–3 and greater than 3 degrees represent low, median and high risk of transmission, respectively.

### Analysis of drug-resistant mutations

All assembled partial *pol* genes were submitted to the Stanford HIV Drug Resistance Database website. TDR mutations were identified using the WHO 2009 list of mutations for surveillance of TDR as implemented in the Calibrated Population Resistance tool (v5.0 beta) [16] (http://hivdb.stanford.edu).

## Results

### Demographic features

Demographic information of 486 individuals with successfully obtained *pol* sequences (GenBank MN633443-MN633928) is summarized in Table 1. The average age of those individuals was 38.30 years (ranging from 8 to 84 years). More male individuals (424, 87.24%) were identified than female individuals (58, 11.93%), and the sex of 4 individuals (0.82%) was unknown. The transmission routes were complicated and included MSM (253, 52.06%), heterosexual transmission (210, 43.21%) and blood-borne transmission (1, 0.21%). The transmission routes of the other cases (22, 4.53%) were unknown. Ethnics information was also collected, among which Han people were the majority (460 of 486, 94.65%). Different education levels were observed, including illiterate (29, 5.97%), primary school (63, 12.96%), middle school (142, 29.22%), senior high school and middle special school (84, 17.28%), college or above (144, 29.63%), and unknown (24, 4.94%).

**Table 1:** Social-demographic characteristics of participants

Characteristics	Case number and percentage
Age (years old)	
≤ 20	27 (5.56%)
21–30	159 (32.72%)
31–40	95 (19.55%)
41–50	90 (18.52%)
>50	94 (19.34%)
Unknown	21 (4.32%)
Gender	
Male	424 (87.24%)
Female	58 (11.93%)
Unknown	4 (0.82%)
Route of transmission	
Heterosexual	210 (43.21%)
MSM ^a^	253 (52.06%)
Blood-borne	1 (0.21%)
Unknown	22 (4.53%)
Ethnic group	
Han	460 (94.65%)
Yi	3 (0.62%)
Dong	1 (0.21%)
Miao	1 (0.21%)
Unknown	21 (4.32%)
Marital status	
Divorced or widowed	65 (13.37%)
Single	207 (42.59%)
Married	192 (39.51%)
Unknown	22 (4.53%)
Degree of education	
Illiterate	29 (5.97%)
Primary school	63 (12.96%)
Middle school	142 (29.22%)
Senior high school and middle special school	84 (17.28%)
College or above	144 (29.63%)
Unknown	24 (4.94%)

^a^*MSM* men who have sex with men

### Distribution of HIV-1 genetic forms

**Fig. 2:**
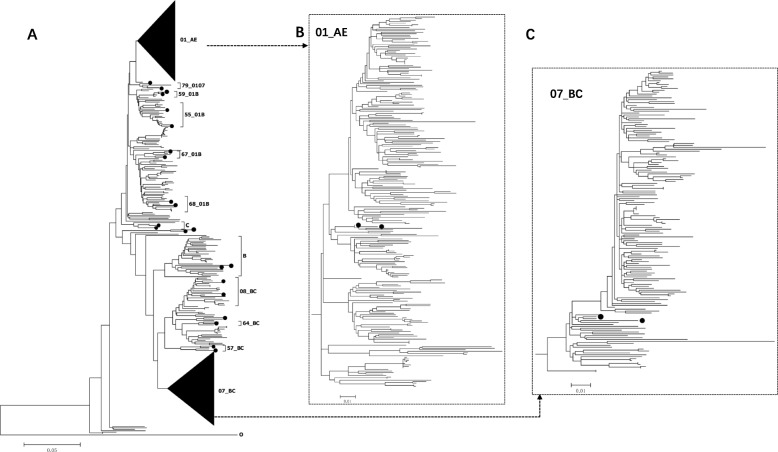
The neighbour-joining phylogenetic tree was built using 486 *pol* sequences obtained from Anhui Province (Fig. 2**a**). Subtype O was used as the outgroup. The subtype reference sequences are marked as solid circles in the tree. Sequences located among different subtypes or CRFs in the NJ tree were URFs. The scale bars representing genetic distance are listed at the bottom. The two black triangles in the Fig. 2**a** represent the sequences of CRF01_AE and CRF07_BC, and the corresponding phylogenetic trees are shown in Figs. 2**b** and **c**

The subtypes of 486 HIV strains with *pol* sequences were determined as described in the Materials and Methods. Multiple subtypes were identified (Fig. 1c), including CRF01_AE (226, 46.50%), CRF07_BC (151, 31.07%), 08_BC (20, 4.12%), 55_01B (15, 3.09%), 57_BC (2, 0.41%), 59_01B (2, 0.41%), 64_BC (1, 0.21%), 67_01B (3, 0.62%), 68_01B (7, 1.44%), 79_0107 (2, 0.41%), B (28, 5.76%), C (2, 0.41%). URFs with genomic sequences of 01B (9, 1.85%), 01 BC (4, 0.82%), 01C (6, 1.23%) and BC (8, 1.65%) were also identified. In the phylogenetic tree, sequences belonging to different subtypes clustered separately as expected. Furthermore, several clusters supported by high bootstrap values were also found among different subtypes, suggesting that various founder viruses were introduced into populations separately (Fig. 2). Diverse subtypes were also observed in populations with the same transmission route. Among the 253 strains from MSM, 121 were 01_AE (47.83%), 90 were 07_BC (35.57%), 10 were 55_01B (3.95%), 10 were B (3.95%), 4 were 68_01B (1.58%), 2 were 67_01B (0.79%), 1 were 08_BC (0.40%), 1 were 79_0107 (0.40%), 1 were C (0.40%), and 13 were URFs (5.14%). Similar subtype distributions of HIV subtypes were also observed in people infected with HIV through heterosexual transmission. In both populations infected through MSM and heterosexual transmission, CRF01_AE was the dominant strain.

### Transmission network analysis

**Fig. 3:**
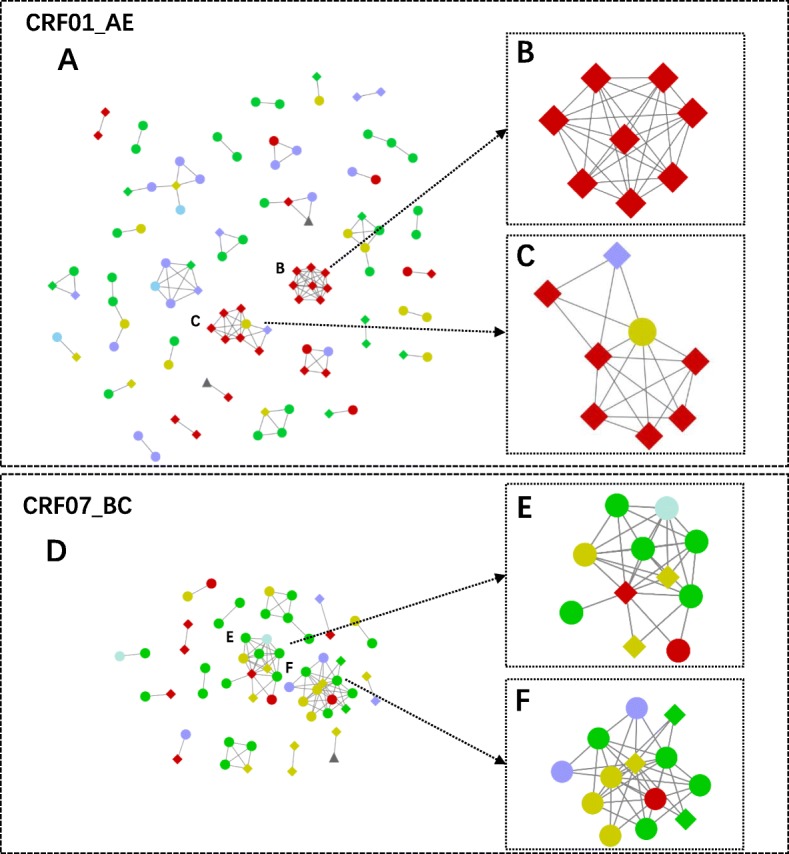
Transmission networks of CRF01_AE (Fig. 3a) and CRF07_BC strains (Fig. 3d). The node represents an HIV sequence or an HIV-infected person, and the edge represents two HIV-infected people connected with a potential transmission relationship. The diamonds, ellipses and triangles represent individuals infected by HIV through heterosexual contact, homosexual contact or unknown contact, respectively. The 2 subnetworks of CRF01_AE (Fig. 3b, Fig. 3c ) and the other 2 subnetworks of CRF07_BC (Fig. 3e, Fig. 3f) on the right contain sequences from patients with higher degrees. The different colours represent different ages. Blue, green, yellow, purple and red represent ages ≤20, 21–30, 31–40, 41–50, and > 50, respectively

Considering that CRF01_AE and CRF07_BC were the two most dominant strains circulating in Anhui Province, we chose 226 sequences of CRF01_AE and 150 of CRF07_BC to construct a transmission network for further analysis as described in the Materials and Methods. For the construction of a transmission network of CRF01_AE sequences (Fig. 3a), a genetic distance ≤0.015 was selected as the cutoff value. A total of 100 nodes, 119 edges and 34 subnetworks with potential transmission relationships were finally identified. The mean age of individuals containing sequences in the CRF01_AE networks was 41.66. Among them, the mean age of the MSM population with sequences in networks was 33.63, which is much younger than the mean age of the heterosexual transmission population (52.38). This result means that young MSMs might be more likely to be involved in transmission. The proportion of individuals with high transmission risk (degree≥4) is 21.00% (21/100). Two subnetworks containing more than 6 individuals were identified and submitted for further analysis. The degree of most nodes in these two subnetworks was higher than 3, indicating a high risk of transmission. The average ages of individuals in these two networks were 67.88 (Fig. 3b) and 61.50 (Fig. 3c).

For the construction of the transmission network of CRF07_BC strains (Fig. 3d), a genetic distance ≤0.014 was selected as the cutoff value. Fifty-seven nodes, 95 edges and 16 subnetworks were identified. The mean age of individuals involved in the network was 35.02. Among them, the mean age of the MSM population was 31.03, while that of heterosexually transmitted people was 43.44. The proportion of high transmission risk people (degree ≥4) is 31.58% (18/57). Two subnetworks containing more than 6 nodes were identified for further analysis. People involved in these two subnetworks were mainly composed of MSMs (Fig. 3e, f). The average ages of the individuals in the two subnetworks were 31.73 and 34.15.

### Transmitted drug-resistant mutations

The prevalence rate of transmitted drug resistance in individuals for different classes of drugs is shown in Table 2. Among 486 strains, 2 strains (0.41%) contained mutations associated with drug resistance to both nucleoside reverse transcriptase inhibitors (NRTIs) and nonnucleoside reverse transcriptase inhibitors (NNRTIs). One strain (0.21%) contained mutations conferred to NRTIs. Twelve strains (2.47%) contained drug resistance mutations related to NNRTIs. In total, the prevalence rate of TDR in Anhui Province was 3.09%. The most commonly observed NNRTI resistance-associated mutations were K103KN/N (8/12, 66.67%) and V106I/VI (3/12, 25.00%).

**Table 2:** The proportion of drug-resistance in individuals according to drug classes

Drug classes ^a^	Cases	Proportion
NRTIs	1	0.21%
NNRTIs	12	2.47%
Dual resistance to NRTIs and NNRTIs	2	0.41%
Susceptible	471	96.91%
Total	486	100.00%

Note: ^a^*NRTIs* nucleoside reverse transcriptase inhibitors, *NNRTIs* non-nucleoside reverse transcriptase inhibitors; the drug classes were exclusive of each other in the study population

## Discussion

Originally, the dominant transmission route of HIV in Anhui Province was paid blood donation [3]. In this study, we found that the MSM population (253, 52.06%) has become the dominant transmission route in newly reported HIV-positive cases, which demonstrated that the high-risk population of HIV infection in the area had switched significantly. Due to social discrimination and cultural stigma associated with homosexual behaviour in China, an estimated 31.5% of HIV-positive MSM have sex with both men and women within and out of marriage [17]. Hence, the spread of HIV in MSM will accelerate the HIV epidemic in the area. More interventions will be necessary for HIV prevention in the specific population.

A high number of URFs was found in Anhui Province (5.55%, 27/486), indicating that many individuals were repeatedly infected by different HIV strains. Many URFs exhibited divergent recombination patterns with mosaic pieces of diverse subtypes in the whole genome [18]. The frequent recombination of the HIV genome will accelerate the evolution of HIV strains and contribute to the emergence of HIV strains with high fitness [19]. Therefore, surveillance of HIV strains based on near-full-length genomic sequences will be better for understanding the HIV epidemic in Anhui Province. Given the high level of strain diversity already present in the province, continued monitoring for changes in subtype distribution and continued support for testing, treatment, and prevention programmes will be necessary to maintain control of HIV-1 in Anhui Province [20].

The transmission network analysis can distinguish potential relationships among different HIV-positive individuals. Changes in the network structure of infected people affect the direction of HIV infection [21]. To date, many studies based on transmission network analysis have been implemented. Zhang et al [22] constructed a transmission network to identify individuals with many potential transmission links and to explore the evolutionary dynamics of the virus among men who have sex with men (MSM) in Beijing. Zhang et al [23] demonstrated that cross-regional HIV transmission in MSM was common and identified a key region for HIV transmission by using a transmission network. Most studies on transmission network analysis were fulfilled in the MSM population. In this study, we identified two subnetworks of CRF01_AE that contained individuals with a high risk of transmission in a heterosexually transmitted population. This result indicated that heterosexual transmission of CRF01_AE strains might play an important role in the local HIV epidemic. Among the 486 individuals, 463 (95.27%) were infected by sexual transmission, which was the main route of HIV-1 transmission in Anhui Province. In these two subnetworks, 16 were sexually transmitted infections. Therefore, we could exclude transmission routes other than sexual transmission. Furthermore, most of the individuals involved in these two subnetworks were elderly people, who were always considered to have a low risk of HIV transmission because of less sexual activity and seldom move among different areas. Several articles have reported that older people also remain sexually active and exhibit a moderate to high level of interest towards sex well into their 80s [24–26]. Moreover, they were even more likely to have multiple partners and use condoms inconsistently [27]. In the two transmission subnetworks in this study, 16 individuals were infected by the sexually transmitted route. Therefore, we believe HIV-1 is spreading sexually among these people. Our results based on the transmission network highlight the importance of elderly people in HIV transmission in Anhui Province and the urgent need for the implementation of effective preventive measures to reduce transmission in the population.

The substitution rates differ significantly among HIV-1 subtypes and CRFs [28]. The average genetic distances between sequences belonging to different subtypes vary. Therefore, the genetic distance cutoff value for the construction of the transmission network also varied. In this study, genetic distances ≤0.015 and ≤ 0.014 were set as cutoff values for CRF01_AE and CRF07_BC, respectively. The value is similar to the value always used for construction of the subtype B transmission network [28–30]. To date, very few studies have been performed on the CRF07_BC transmission network because very small genetic distances were always found between CRF07_BC strains from different individuals. In this study, 0.014 was set as the cutoff value for the construction of the CRF07_BC transmission network based on maximizing the number of transmission clusters, which is similar to that for CRF01_AE. However, it should not be used comprehensively for CRF07_BC strains. The cutoff value for the construction of a transmission network based on strains prevalent in different areas or populations should be tested every time.

The “Four Free and One Care” project was initiated in China in 2003. Since then, HAART has been widely used, and increasing levels of HIV drug resistance in some specific areas or populations have been revealed [31]. In this study, we found that the prevalence of HIV drug resistance (3.09%) was lower than the WHO HIVDR surveillance threshold of 5%. Shen et al [9] researched the prevalence of transmitted HIV drug resistance among MSM in Anhui Province and identified that the two NNRTI-related SDRMs were Y181C and G190A, found in persons infected with CRF01_AE and B strains, respectively. In our study, the most common NNRTI-related SDRMs were K103 N/KN and V106I/VI, found in CRF01_AE, CRF07_BC, CRF08_BC, B, CRF55_01B, CRF59_01B, CRF68_01B and a URF_01B. The results indicated that different prevalence subtypes may lead to different SDRMs.

This study fills an important gap in the extant literature on the transmission network structure and epidemic status in Anhui Province. However, there are limitations. Although the samples in the study were collected from all regions in Anhui Province, the distributions among different districts were unequal. In addition, only one cross-sectional study was conducted, and the results could not be used to observe the dynamics of the local HIV epidemic. Despite these limitations, this is the first report on province-wide exploration of HIV genetic characterizations in Anhui Province.

## Conclusions

In this study, we found that the MSM population (253, 52.06%) has become the dominant transmission route in newly reported HIV-positive cases in Anhui Province, and a high number of URFs (5.55%, 27/486) was found. The transmission networks indicated that heterosexual transmission of CRF01_AE strains might play an important role in the local HIV epidemic, and elderly people should be given more attention. The prevalence of HIV drug resistance (3.09%) was lower than the WHO HIVDR surveillance threshold of 5%. Our results will provide information for the further investigation and research of HIV epidemics in the area. The discovery of the characteristics of individuals involved in high-risk transmission will provide a target population for behavioural intervention of HIV and help further investigations of HIV transmission in Anhui Province.

## Data Availability

All data and materials described in the manuscript are available.

## Abbreviations

AIDS: Acquired immunodeficiency syndrome
ART: Antiretroviral therapy
CRFs: Circulating recombinant forms
HAART: Highly active antiretroviral therapy
HIV: Human immunodeficiency viruses
MSM: Men who have sex with men
NNRTIs: Nonnucleoside reverse transcriptase inhibitors
NRTIs: Nucleoside reverse transcriptase inhibitors
ORFs: Open reading frames
SDRMs: Surveillance/transmitted drug resistance mutations
TDR: Transmitted drug resistance
TN93: Tamura-Nei 93
URFs: Unique recombinant forms
